# Too much tolerance for hyperoxemia in mechanically ventilated patients with SARS-CoV-2 pneumonia? Report from an Italian intensive care unit

**DOI:** 10.3389/fmed.2022.957773

**Published:** 2022-07-28

**Authors:** Elisa Damiani, Erika Casarotta, Andrea Carsetti, Giulia Mariotti, Sara Vannicola, Rachele Giorgetti, Roberta Domizi, Claudia Scorcella, Erica Adrario, Abele Donati

**Affiliations:** ^1^Department of Biomedical Sciences and Public Health, Università Politecnica delle Marche, Ancona, Italy; ^2^Anesthesia and Intensive Care Unit, Azienda Ospedaliera Universitaria “Ospedali Riuniti Umberto I-Lancisi-Salesi”, Ancona, Italy

**Keywords:** COVID-19, SARS-CoV-2, ARDS, hyperoxia, oxygen, ventilator-associated pneumonia

## Abstract

**Background:**

In COVID-19 patients requiring mechanical ventilation, the administration of high oxygen (O_2_) doses for prolonged time periods may be necessary. Although life-saving in most cases, O_2_ may exert deleterious effects if administered in excessive concentrations. We aimed to describe the prevalence of hyperoxemia and excessive O_2_ administration in mechanically ventilated patients with SARS-CoV-2 pneumonia and determine whether hyperoxemia is associated with mortality in the Intensive Care Unit (ICU) or the onset of ventilator-associated pneumonia (VAP).

**Materials and methods:**

Retrospective single-center study on adult patients with SARS-CoV-2 pneumonia requiring invasive mechanical ventilation for ≥48 h. Patients undergoing extracorporeal respiratory support were excluded. We calculated the excess O_2_ administered based on the ideal arterial O_2_ tension (PaO_2_) target of 55–80 mmHg. We defined hyperoxemia as PaO_2_ > 100 mmHg and hyperoxia + hyperoxemia as an inspired O_2_ fraction (FiO_2_) > 60% + PaO_2_ > 100 mmHg. Risk factors for ICU-mortality and VAP were assessed through multivariate analyses.

**Results:**

One hundred thirty-four patients were included. For each day of mechanical ventilation, each patient received a median excess O_2_ of 1,121 [829–1,449] L. Hyperoxemia was found in 38 [27–55]% of arterial blood gases, hyperoxia + hyperoxemia in 11 [5–18]% of cases. The FiO_2_ was not reduced in 69 [62–76]% of cases of hyperoxemia. Adjustments were made more frequently with higher PaO_2_ or initial FiO_2_ levels. ICU-mortality was 32%. VAP was diagnosed in 48.5% of patients. Hyperoxemia (OR 1.300 95% CI [1.097–1.542]), time of exposure to hyperoxemia (OR 2.758 [1.406–5.411]), hyperoxia + hyperoxemia (OR 1.144 [1.008–1.298]), and daily excess O_2_ (OR 1.003 [1.001–1.005]) were associated with higher risk for ICU-mortality, independently of age, Sequential Organ failure Assessment score at ICU-admission and mean PaO_2_/FiO_2_. Hyperoxemia (OR 1.033 [1.006–1.061]), time of exposure to hyperoxemia (OR 1.108 [1.018–1.206]), hyperoxia + hyperoxemia (OR 1.038 [1.003–1.075]), and daily excess O_2_ (OR 1.001 [1.000–1.001]) were identified as risk factors for VAP, independently of body mass index, blood transfusions, days of neuromuscular blocking agents (before VAP), prolonged prone positioning and mean PaO_2_/FiO_2_ before VAP.

**Conclusion:**

Excess O_2_ administration and hyperoxemia were common in mechanically ventilated patients with SARS-CoV-2 pneumonia. The exposure to hyperoxemia may be associated with ICU-mortality and greater risk for VAP.

## Introduction

Supplemental oxygen (O_2_) is a life-saving therapy in hypoxemic patients in order to guarantee adequate tissue O_2_ delivery. Nonetheless, excessive O_2_ administration may also exert deleterious effects (1). In recent years, several studies supported the use of more conservative oxygenation strategies in Intensive Care Units (ICUs), whereas liberal O_2_ therapy and the exposure to arterial hyperoxia in critically ill patients were associated with adverse outcomes (2–4). The lung is the first organ affected by O_2_ toxicity. Hyperoxia induces oxidative stress and inflammation in the lung (1) and may impair the surfactant system, thus causing alveolar collapse and the reduction in pulmonary compliance (5). Excess O_2_ administration may also compromise muco-ciliary clearance and the anti-microbial capacity of the immune cells, thus contributing to the development of ventilator-associated pneumonia (VAP) (6).

In moderate or severe acute respiratory distress syndrome (ARDS), the administration of high inspired O_2_ fractions (FiO_2_) is frequently required to maintain normoxemia (arterial O_2_ tension [PaO_2_] 80–100 mmHg) and this may predispose to additional hyperoxia-induced lung injury. In order to limit the exposure to hyperoxia, the ARDS Network recommends using a PaO_2_ target of 55–80 mmHg in mechanically ventilated patients (7). Nonetheless, ARDS patients are frequently exposed to excessive FiO_2_ levels (8) and even undergo a condition of hyperoxemia (PaO_2_ > 100 mmHg) in a substantial number of cases (9). In a meta-analysis of randomized controlled trials (RCTs), excessive O_2_ administration (FiO_2_ > 50%) resulting in PaO_2_ levels above the protocol goal (>80 mmHg) was associated with mortality and lower ventilator- and hospital-free days (10). A recent multicentre RCT was aimed to compare a conservative (target PaO_2_ 55–70 mmHg, SpO_2_ 88–92%) with a liberal oxygenation strategy (target PaO_2_ 90–105 mmHg, peripheral O_2_ saturation [SpO_2_] ≥ 96%) in ARDS patients, however, this study was prematurely stopped due to safety concerns (higher mortality and five mesenteric ischemic events in the conservative O_2_ group) (11). Therefore, which is the safest oxygenation target for mechanically ventilated patients with ARDS remains an open question.

This is a problem of major importance for patients with severe acute respiratory syndrome due to novel Coronavirus (SARS-CoV-2). A large Italian cohort study showed that 12% of these patients received FiO_2_ up to 100% and an FiO_2_ ≥ 50% was necessary in 89% of total (12). Hyperoxia-induced lung injury may add to the inflammatory process caused by the viral infection. Moreover, these patients often require long periods of mechanical ventilation and are at risk of exposure to high O_2_ concentrations for several days (13). Despite the key role of O_2_ in the treatment of SARS-CoV-2, the potential adverse effects of a prolonged exposure to hyperoxia in this patient category remain unexplored.

The primary aim of this study was to explore whether mechanically ventilated patients with SARS-CoV-2 pneumonia in our ICU received an excessive amount of O_2_ and were exposed to hyperoxemia. In addition, we evaluated if hyperoxemia was associated with mortality or the onset of VAP.

## Materials and methods

This retrospective observational study was conducted in the “General, respiratory and major trauma Intensive care Unit” of the Azienda Ospedaliera Universitaria “Ospedali Riuniti Umberto I-Lancisi-Salesi” of Ancona, Italy. During the pandemic phase, this ICU provided 18 beds for COVID-19 patients. The study protocol was approved by the local Ethics Committee (Comitato Etico Regionale Marche). Written informed consent was not requested due to the retrospective study design. This study included all consecutive adult (>18 year old) patients with SARS-CoV-2 pneumonia admitted to the ICU between February 2020 and May 2021, who required endotracheal intubation and invasive mechanical ventilation for at least 48 consecutive hours. COVID-19 infection was confirmed by means of real time polymerase chain reaction on nasopharyngeal swab or bronchoalveolar lavage. Exclusion criteria were: duration of mechanical ventilation < 48 h; ICU-discharge or death within 48 h; use of extracorporeal membrane oxygenation (ECMO) or extracorporeal carbon dioxide removal (ECCO_2_R); admission from another ICU with a length of stay > 48 h; re-admissions after a previous ICU admission for SARS-CoV-2 pneumonia; COVID-19+ patients without pneumonia admitted to the ICU for different reasons.

### Patient management

According to the most recent guidelines for the management of ARDS and COVID-19 (7, 14, 15), all patients received a lung protective ventilation strategy with a tidal volume (TV) of 4–6 ml/kg of ideal body weight, while maintaining a plateau pressure (Pplat) ≤ 30 cmH_2_O and a driving pressure ≤ 15 cmH_2_O. A positive end-expiratory pressure (PEEP) ≥ 5 cmH_2_O was applied in all patients, using an open-lung strategy. Neuromuscular blocking agents (NMBA) (continuous infusion of cisatracurium or rocuronium) were used in the early phase of mechanical ventilation in cases of refractory hypoxemia despite deep sedation to facilitate lung protective ventilation, during prone positioning, in cases of patient-ventilator dyssynchrony and/or in presence of high respiratory drive despite optimal sedation (15). Prone positioning was used in patients with a PaO_2_/FiO_2_ < 150 mmHg for a duration of at least 16 h per session (15). During the COVID-19 pandemic, we implemented and applied a protocol of prolonged prone positioning with a duration of 36 consecutive hours per session, which showed to be feasible and potentially more effective in improving oxygenation (16). Inhaled nitric oxide was administered as a rescue therapy in cases of refractory hypoxemia despite the use of NMBA and prone ventilation. Veno-venous ECMO was used in patients with refractory hypoxemia despite all other therapies (17).

A VAP prevention strategy was routinely applied, including: oral care with chlorhexidine three times a day; head of bed elevation (30° whenever possible); check (3 times a day) and maintenance of the cuff pressure of the endotracheal tube (25 cmH_2_O); routine aspiration of tracheal secretions by means of a closed suction system; frequent check of the correct positioning and function of the nasogastric tube; weaning from mechanical ventilation as soon as possible; tracheostomy in cases of anticipated difficult weaning (18). During prone ventilation, the administration of enteral nutrition was discontinued or, alternatively, continued at low dosage (10–20 ml/h) with frequent check of the gastric residual volume and possible episodes of regurgitation. Stress ulcer prophylaxis (pantoprazole 40 mg/day) was administered in patients with risk factors for gastro-intestinal bleeding (including coagulopathy, mechanical ventilation >48 h, hypotension), who represented the vast majority of the patients enrolled. Deep venous thrombosis prophylaxis was applied in all patients with low molecular weight heparin (at least 4,000 UI/day). In patients with VAP, antibiotic therapy was based on local written protocols in accordance with the most recent guidelines (19).

### Data collection

For each included patient we collected: age, gender, body mass index (BMI), comorbidities (obesity, arterial hypertension, diabetes mellitus, chronic obstructive pulmonary disease, ischemic cardiomyopathy), the Sequential Organ Failure Assessment (SOFA) score at ICU admission, arterial lactate levels at ICU admission, PaO_2_/FiO_2_ at ICU admission, ICU length-of-stay, duration of invasive mechanical ventilation, ICU mortality, antibiotics prior to ICU admission, steroids prior to ICU admission, total steroid dose administered in the ICU (adjusted for body weight, mg/kg/day), episodes of VAP (early/late onset, causal pathogen, antibiotic resistance), use of NMBA, prone positioning (number of sessions, prolonged prone ventilation), use of proton pump inhibitors, blood transfusions (number of units).

The onset of VAP was defined by the detection of a causative agent in a respiratory sample (tracheal aspirate or bronchoalveolar lavage) associated with a new or progressive lung infiltrate, plus at least two clinical criteria (fever or hypothermia, leukocytosis or leukopenia, purulent secretions), after at least 48 h of invasive mechanical ventilation (18). In cases of multiple VAP episodes, only the first episode was considered. An episode of VAP occurring within 4 days after endotracheal intubation was defined as “early onset VAP” (18).

Starting from endotracheal intubation and for the whole duration of invasive mechanical ventilation, we collected data from all arterial blood gas (ABG) analyses, including PaO_2_, SaO_2_, pH, PaCO_2_ with date and time. In our ICU, ABG are generally performed at least every 8 h in all patients. For each ABG we recorded data on mechanical ventilation: TV (ml), respiratory rate (RR), minute ventilation (l/min), PEEP, FiO_2_, and PaO_2_/FiO_2_. In addition, for each ABG we noted if the patient was on NMBA, prone ventilation, and measures of respiratory mechanics if available (Pplat, driving pressure, static compliance [Cstat]).

Based on the ARDS Network protocol, we considered a PaO_2_ of 80 mmHg as the upper limit of the oxygenation target. For each ABG showing a PaO_2_ > 80 mmHg, we estimated the “ideal FiO_2_” that would be sufficient to obtain a PaO_2_ of 80 mmHg based on the PaO_2_/FiO_2_ ratio, according to the following formula:


$${\mathrm{FiO}}_{2ideal}=\left(80*{\mathrm{FiO}}_{2actual}\right)/{\mathrm{PaO}}_{2actual}$$

We then estimated the excess O_2_ being administered as follows:


ExcessO(L/min)2=Minuteventilation(L/min)*(FiO2 actual-FiO2 ideal)

We then multiplied the value obtained for the time (minutes) elapsed since the previous ABG and calculated:

- Total excess O_2_ (L): total cumulative amount of excess O_2_ administered during the whole duration of invasive mechanical ventilation;
- Daily excess O_2_ (L/day): average amount of excess O_2_ administered in each day of invasive mechanical ventilation;
- Three-days excess O_2_ (L): cumulative amount of excess O_2_ administered during the first 3 days of invasive mechanical ventilation;
- Daily excess O_2_ before VAP (L/day): average amount of excess O_2_ administered in each day of invasive mechanical ventilation before the first diagnosis of VAP.

Episodes of “hyperoxemia” were defined by a PaO_2_ > 100 mmHg. We defined as “uncorrected hyperoxemia” any episode of hyperoxemia that was not followed by a reduction in the FiO_2_. In addition, we defined as “hyperoxia + hyperoxemia” any episode of FiO_2_ > 60% in presence of a PaO_2_ > 100 mmHg. Herein, the term “hyperoxia” (generally defined as the administration of any O2 dose > 21%) was used to indicate a high FiO2 (>60%). We then calculated the prevalence of hyperoxemia and hyperoxia + hyperoxemia for the whole duration of invasive mechanical ventilation, in the first 3 days of mechanical ventilation and for the days prior to VAP diagnosis. The duration of exposure to hyperoxemia was estimated as follows:

1. Daily time of exposure to hyperoxemia (hours per day of MV): whenever an ABG showed a PaO2 > 100 mmHg, we considered as time of exposure to hyperoxemia the time elapsed since the previous ABG; the total sum of hours was then divided by the number of days of MV.
2. Time of exposure to hyperoxemia in the first 3 days of MV: total sum of hours of hyperoxemia in the first 3 days of MV.
3. Daily time of exposure to hyperoxemia before VAP: average amount of hours of hyperoxemia in each day of invasive mechanical ventilation before the first diagnosis of VAP.

In addition, we calculated: mean FiO_2_, mean PaO_2_, mean PaO_2_/FiO_2_, highest FiO_2_, highest PaO_2_.

### Statistical analysis

Statistics was performed with GraphPad Prism version 6 (GraphPad Software, La Jolla, CA, United States) and Statistical Package for Social Science software, version 17.0 (SPSS Inc., Chicago, IL, United States). Normality of distribution was checked with the Shapiro–Wilk test. Continuous variables were expressed as mean ± standard deviation or median [1*^st^*–3*^rd^* quartile], as appropriate. Unpaired *t*-test or Mann Whitney *U*-test were used for comparisons of two groups. The chi-square test was used for nominal variables. We constructed multivariate binary logistic regression models in order to evaluate the independent association between the exposure to hyperoxemia (prevalence of hyperoxemia, prevalence of hyperoxia + hyperoxemia, daily excess O_2_) and the outcomes of interest (ICU mortality, diagnosis of VAP). Separate models were constructed for each index of exposure to hyperoxemia in order to avoid multi-collinearity. The basic assumptions for conducting logistic regression analyses were verified, including the absence of multi-collinearity and the linearity of the logit for each continuous independent variable (20). Covariates included in the models were selected based on their well-established association with the outcome of interest (6, 20). A *p*-value < 0.05 was used to indicate statistical significance.

## Results

Between February 27, 2020 and May 12, 2021, a total of 207 COVID-19 patients was admitted to our ICU. Of these, exclusions were: 43 patients who underwent ECMO or ECCO_2_R; 7 patients who had an ICU length of stay < 48 h; 1 patient who was extubated before 48 h; 1 non-intubated patient; 13 patients transferred from a different ICU in which they stayed for more than 48 h; 6 re-admissions; 2 COVID-19+ patients without pneumonia. Therefore, we included 134 patients in total. The vast majority of patients was male, and the most frequent comorbities were obesity and arterial hypertension (Table 1). ICU-mortality was 32%. ICU Non-survivors were older, had a higher prevalence of ischemic cardiomyopathy and worse PaO_2_/FiO_2_ and SOFA score at ICU-admission (Table 1).

**TABLE 1:** General characteristics of the included patients.

	All patients (*n* = 134)	ICU survivors (*n* = 91, 68%)	ICU non-survivors (*n* = 43, 32%)	*p*
Age (years)	66 [57–74]	62 [55–72]	72 [66–75]	0.0004
Gender (n, % of males)	106 (79%)	72 (68%)	34 (32%)	0.995
Body mass index	29 [26–33]	29 [26–34]	28 [24–33]	0.138
Comorbidities (n, %)				
Arterial hypertension	71 (53%)	44 (62%)	27 (38%)	0.118
Obesity	52 (39%)	36 (69%)	16 (31%)	0.794
Diabetes mellitus	22 (16%)	17 (77%)	5 (23%)	0.303
Ischemic cardiomyopathy	15 (11%)	6 (40%)	9 (60%)	0.014
Chronic obstructive pulmonary disease	6 (4.5%)	4 (67%)	2 (33%)	0.947
Immunosuppression	2 (1.5%)	1 (1%)	1 (2.3%)	0.540
ICU length of stay (days)	17 [11–27]	17 [11–29]	16 [9–24]	0.129
Duration of mechanical ventilation (days)	14 [8–26]	14 [8–28]	16 [9–24]	0.859
SOFA score (admission)	7 [6–8]	7 [6–8]	7 [7–9]	0.0029
Lactate levels (admission, mmol/L)	1.3 [1–1.6]	1.3 [1.1–1.6]	1.3 [1.1–1.7]	0.578
PaO_2_/FiO_2_ (admission, mmHg)	111 [81–173]	122 [90–188]	101 [73–123]	0.0095

ICU, Intensive Care Unit; SOFA, Sequential Organ Failure Assessment.

### Excess O_2_ and hyperoxemia

**FIGURE 1:**
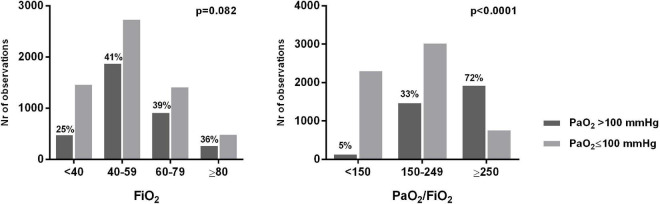
Number and percentage of arterial blood gases showing hyperoxemia stratified by PaO_2_ and FiO_2_ levels.

We analyzed 9,583 ABGs in total. A PaO_2_ > 80 mmHg was found in 68.8% of cases. All the analyzed patients received an excessive amount of O_2_ in relation to the oxygenation target indicated by the guidelines (55–80 mmHg). We estimated that, for the whole duration of invasive mechanical ventilation, each patient received an average excess O_2_ of 17,741 [8,950–27,248] L, corresponding to a daily excess O_2_ of 1,121 [829–1,449] L (minimum: 319 L; maximum: 3,818 L). In each patient, hyperoxemia (PaO_2_ > 100 mmHg) was present in 38 [27–55]% of ABGs, and 11 [5–18]% of ABGs showed a condition of hyperoxia + hyperoxemia (FiO_2_ > 60% + PaO_2_ > 100 mmHg). Hyperoxemia was more frequently associated with higher PaO_2_/FiO_2_ and possible indicators of less severe pulmonary dysfunction (lower Pplat and driving pressure) (Table 2). Figure 1 show the prevalence of hyperoxemia stratified by the FiO_2_ and PaO_2_/FiO_2_.

**TABLE 2:** Comparison of all arterial blood gases with or without hyperoxemia (PaO_2_ > 100 mmHg).

	Hyperoxemia (PaO_2_ > 100 mmHg, *n* = 3,517)	No hyperoxemia (PaO_2_ ≤ 100 mmHg, *n* = 6,066)	*p*
PaO_2_ (mmHg)	120 [109–140]	81 [71–90]	<0.0001
SaO_2_ (%)	99.4 [99.1–99.9]	98.5 [97.3–99.4]	<0.0001
FiO_2_ (%)	50 [40–60]	50 [40–60]	<0.0001
PaO_2_/FiO_2_ (mmHg)	257 [211–302]	170 [128–220]	<0.0001
Ventilation mode (n, %)			<0.0001
Volume controlled	890 (25%)	803 (13%)	
Pressure-controlled ventilation-volume guaranteed	1,091 (31%)	1,460 (24%)	
Pressure controlled	586 (17%)	1,207 (20%)	
Pressure support	767 (22%)	2,329 (38%)	
Continuous positive airway pressure	91 (2%)	93 (2%)	
Spontaneous breathing	92 (3%)	174 (3%)	
Minute ventilation (L/min)	9 [8–10.5]	9.1 [8–11]	0.0002
Peep (cmH_2_O)	10 [10–12]	10 [8–12]	<0.0001
pH	7.44 [7.39–7.48]	7.45 [7.39–7.48]	<0.0001
PaCO_2_ (mmHg)	45 [40–51]	45 [40–52]	<0.0001
Cstat (mL/cmH_2_O)	47 [38–56]	42 [33–53]	<0.0001
Plateau pressure (cmH_2_O)	23 [21–25]	24 [22–26]	<0.0001
Driving pressure (cmH_2_O)	11 [9–13]	12 [10–14]	<0.0001

PEEP, Positive End-Expiratory Pressure; Cstat, Static compliance.

**FIGURE 2:**
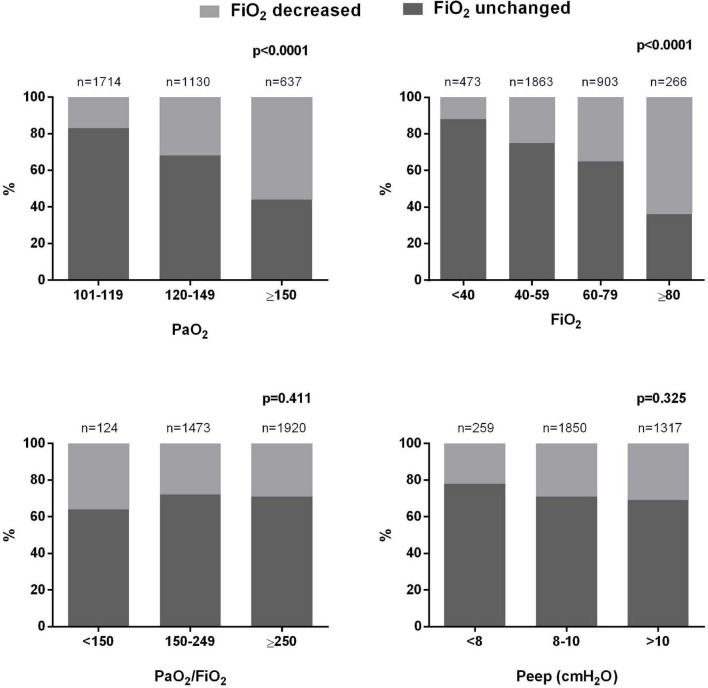
Episodes of uncorrected hyperoxemia stratified by PaO_2_, FiO_2_, PaO_2_/FiO_2_, and PEEP levels.

In 69 [62–76]% of cases, an ABG showing hyperoxemia was not followed by a reduction in the FiO_2_ (uncorrected hyperoxemia). The FiO_2_ was more likely to be reduced in presence of higher PaO_2_ and initial FiO_2_ levels, although even in presence of a PaO_2_ ≥ 150 mmHg or an initial FiO_2_ ≥ 80% no change was made in about 40% of cases (Figure 2). The choice of reducing the FiO_2_ was not influenced by PaO_2_/FiO_2_ or PEEP levels (Figure 2).

### Hyperoxemia and intensive care unit-mortality

Intensive care unit-survivors showed higher mean PaO_2_/FiO_2_, mean PaO_2_ and maximum PaO_2_ as compared to Non-survivors, however mean and maximum FiO_2_ were significantly lower (Table 3). Hyperoxemia was more frequent among ICU-survivors, and the daily time of exposure was higher, even if Non-survivors showed a higher prevalence of hyperoxia + hyperoxemia (both for the whole duration of mechanical ventilation and in the first 3 days) (Table 3). After adjusting for age, SOFA score at ICU-admission and mean PaO_2_/FiO_2_, the prevalence of hyperoxemia, the duration of exposure, the prevalence of hyperoxia + hyperoxemia and daily excess O_2_ were independently associated with ICU-mortality (Table 4). The total excess O_2_ and the excess O_2_ in the first 3 days were not significantly associated with mortality in the logistic regression analysis.

**TABLE 3:** Comparison of oxygenation variables between ICU-survivors and Non-survivors.

	ICU-survivors (*n* = 91, 68%)	ICU non-survivors (*n* = 43, 32%)	*p*
Mean PaO_2_ (mmHg)	105 [97–114]	94 [88–100]	<0.0001
Mean FiO_2_ (%)	45 [42–47]	63 [56–69]	<0.0001
Mean PaO_2_/FiO_2_ (mmHg)	242 [222–275]	163 [137–177]	<0.0001
Max PaO_2_ (mmHg)	219 [179–260]	197 [166–227]	0.011
Max FiO_2_ (%)	100 [80–100]	100 [100–100]	0.0007
Hyperoxemia, % of ABGs	44 [30–63]	34 [24–41]	0.0002
Time of exposure to hyperoxemia, hours/day of MV	11 [7–14]	8 [5–10]	0.0006
Uncorrected hyperoxemia, % of ABGs	70 [61–77]	69 [63–75]	0.937
Hyperoxia + Hyperoxemia, % of ABGs	8 [4–14]	17 [11–23]	<0.0001
Hyperoxemia (first 3 days), % of ABGs	64 [50–75]	58 [35–69]	0.066
Time of exposure to hyperoxemia, total hours in the first 3 days	47 [37–59]	44 [26–61]	0.204
Hyperoxia + Hyperoxemia (first 3 days), % of ABGs	20 [13–36]	31 [20–47]	0.0063
Total excess O_2_ (L)	17,449 [8,912–27,118]	19,575 [11,038–29,055]	0.662
Daily excess O_2_ (L)	1,145 [809–1,480]	1,049 [890–1,393]	0.860
Three-days excess O_2_ (L)	6,098 [4,875–7,728]	6,270 [4,531–7,863]	0.689

All variables were calculated for the whole duration of invasive mechanical ventilation unless otherwise specified. ABGs, arterial blood gases.

**TABLE 4:** Binomial logistic regression models for the association between hyperoxemia and ICU-mortality.

	Odds ratio (95% confidence interval)	*p*
**Model 1**		
Hyperoxemia, % of ABGs	1.300 [1.097–1.542]	0.003
Mean PaO_2_/FiO_2_ (mmHg)	0.805 [0.712–0.911]	0.001
SOFA score (ICU admission)	2.043 [0.941–4.435]	0.071
Age (years)	1.034 [0.948–1.127]	0.452
**Model 2**		
Time of exposure to Hyperoxemia, hours/day of MV	2.758 [1.406–5.411]	0.003
Mean PaO_2_/FiO_2_ (mmHg)	0.773 [0.658–0.907]	0.002
SOFA score (ICU admission)	1.892 [0.898–3.985]	0.094
Age (years)	1.041 [0.949–1.141]	0.396
**Model 3**		
Hyperoxemia (first 3 days), % of ABGs	1.077 [1.022–1.135]	0.005
Mean PaO_2_/FiO_2_ (mmHg)	0.877 [0.822–0.935]	<0.001
SOFA score (ICU admission)	1.775 [0.906–3.477]	0.095
Age (years)	1.014 [0.931–1.105]	0.744
**Model 4**		
Time of exposure to Hyperoxemia, total hours in the first 3 days	1.074 [1.017–1.135]	0.010
Mean PaO_2_/FiO_2_ (mmHg)	0.879 [0.825–0.936]	<0.001
SOFA score (ICU admission)	1.779 [0.990–3.194]	0.054
Age (years)	1.007 [0.928–1.093]	0.863
**Model 5**		
Hyperoxia + Hyperoxemia, % of ABGs	1.144 [1.008–1.298]	0.037
Mean PaO_2_/FiO_2_ (mmHg)	0.916 [0.878–0.956]	<0.001
SOFA score (ICU admission)	1.393 [0.800–2.424]	0.241
Age (years)	0.997 [0.931–1.068]	0.933
**Model 6**		
Daily excess O_2_ (L)	1.003 [1.001–1.005]	0.008
Mean PaO_2_/FiO_2_ (mmHg)	0.889 [0.840–0.940]	<0.001
SOFA score (ICU admission)	1.384 [0.790–2.422]	0.256
Age (years)	1.002 [0.932–1.076]	0.964

All variables were calculated for the whole duration of invasive mechanical ventilation, unless indicated otherwise.

Model 1: This model was statistically significant with a χ^2^ (df 4) = 141.754 and *p* < 0.0001. This model explains 91.3% (Nagelkerke *R*^2^) of variance for the outcome ICU-mortality and correctly classifies 96.3% of cases.

Model 2: This model was statistically significant with a χ^2^ (df 4) = 142.335 and *p* < 0.0001. This model explains 91.5% (Nagelkerke *R*^2^) of variance for the outcome ICU-mortality and correctly classifies 95.5% of cases.

Model 3: This model was statistically significant with a χ^2^ (df 4) = 131.543 and *p* < 0.0001. This model explains 87.5% (Nagelkerke *R*^2^) of variance for the outcome ICU-mortality and correctly classifies 93.3% of cases.

Model 4: This model was statistically significant with a χ^2^ (df 4) = 128.237 and *p* < 0.0001. This model explains 87.5% (Nagelkerke *R*^2^) of variance for the outcome ICU-mortality and correctly classifies 93.3% of cases.

Model 5: This model was statistically significant with a χ^2^ (df 4) = 124.466 and *p* < 0.0001. This model explains 86.2% (Nagelkerke *R*^2^) of variance for the outcome ICU-mortality and correctly classifies 92.5% of cases.

Model 6: This model was statistically significant with a χ^2^ (df 4) = 127.466 and *p* < 0.0001. This model explains 85.8% (Nagelkerke *R*^2^) of variance for the outcome ICU-mortality and correctly classifies 93.3% of cases.

ABGs, arterial blood gases; SOFA, Sequential Organ Failure Assessment.

### Hyperoxemia and ventilator-associated pneumonia

Sixty-five patients (48.5% of total) had at least one episode of VAP during the ICU-stay. The vast majority (91%) of VAP episodes was late-onset. The most frequently isolated pathogens were: Staphylococcus Aureus (29 cases); Acinetobacter Baumanii (20 cases); Enterobacteriaceae (*Klebsiella* spp., *Escherichia Coli, Serratia* spp., *Enterobacter* spp., 34 cases); Pseudomonas Aeruginosa (14 cases); Corynebacterium Striatum (10 cases); Proteus Mirabilis (10 cases); Streptococcus Pneumoniae (3 cases); Aspergillus spp. (7 cases). Multi-drug resistant pathogens were isolated in 33% of cases.

Patients with VAP had longer ICU-stay, more frequently underwent prolonged prone positioning, and showed lower PaO_2_/FiO_2_ than those with no episodes of VAP (Table 5). Patients with at least one episode of VAP showed higher prevalence of hyperoxia + hyperoxemia (before VAP and in the first 3 days of mechanical ventilation) and received a higher excess O_2_ (daily and in the first 3 days of mechanical ventilation) (Table 5).

**TABLE 5:** Comparison between patients whit at least one episode of VAP and those without any episode of VAP.

	No VAP (*n* = 69, 51.5%)	VAP (*n* = 65, 48.5%)	*p*
Age (years)	66 [55–73]	67 [58–74]	0.256
Gender (n, % of males)	56 (81%)	50 (77%)	0.547
BMI (kg/m^2^)	28 [25–32]	29 [27–35]	0.056
ICU length of stay (days)	12 [8–17]	25 [19–36]	<0.0001
Days of mechanical ventilation (before VAP)	9 [6–14]	8 [6–12]	0.415
SOFA score (ICU admission)	7 [6–8]	7 [6–8]	0.245
PaO_2_/FiO_2_ (ICU admission, mmHg)	130 [91–193]	101 [72–133]	0.003
Antibiotics before ICU admission (n, %)	29 (42%)	20 (31%)	0.176
Steroids before ICU admission	40 (58%)	40 (61%)	0.674
Steroids in ICU, mg/kg/die	0.06 [0.03–0.09]	0.06 [0.05–0.08]	0.282
NMBA (days before VAP)	3 [1–6]	4 [3–6]	0.143
Prone positioning (number of sessions before VAP)	2 [1–3]	2 [1–3]	0.056
Prolonged prone positioning, number of patients (%)	43 (62%)	55 (85%)	0.004
RBC before VAP (number of units)	0 [0–2]	0 [0–2]	0.753
Mean PaO_2_ (mmHg)	105 [94–116]	107 [99–116]	0.348
Mean FiO_2_ (%)	46 [41–55]	53 [48–59]	<0.001
Mean PaO_2_/FiO_2_ (mmHg)	244 [182–281]	211 [185–238]	0.004
Max PaO_2_ (mmHg)	206 [162–249]	196 [173–249]	0.872
Max FiO_2_ (%)	100 [75–100]	80 [60–100]	0.099
Hyperoxemia (% of ABGs)	47 [27–65]	45 [38–60]	0.492
Time of exposure to hyperoxemia, hours/day before VAP	11 [6–16]	12 [9–15]	0.285
Uncorrected hyperoxemia (% of episodes)	68 [59–76]	69 [55–75]	0.587
Hyperoxia + hyperoxemia (% of ABGs)	11 [6–19]	18 [9–29]	0.002
Hyperoxemia (first 3 days, % of ABGs)	64 [46–75]	60 [48–72]	0.425
Time of exposure to hyperoxemia, total hours in the first 3 days	47 [31–60]	47 [40–59]	0.664
Hyperoxia + Hyperoxemia (first 3 days, % of ABGs)	23 [13–34]	30 [17–42]	0.016
Daily excess O_2_ (L/day)	1,209 [781–1,533]	1,443 [1,154–1,952]	0.001
Three-days excess O_2_ (L)	5,642 [3,915–7,763]	6,343 [5,161–7,763]	0.048

VAP, ventilator-associated pneumonia; BMI, body mass index; ICU, Intensive Care Unit; SOFA, Sequential Organ Failure Assessment; NMBA, neuromuscular blocking agents; RBC, red blood cells; ABGs, arterial blood gases.

After adjusting for BMI, blood transfusions, days of NMBA (before VAP), prolonged prone positioning and mean PaO_2_/FiO_2_ before VAP, a higher prevalence of hyperoxemia was associated with a higher risk of VAP (Table 6): the adjusted risk for VAP increased by 3.3% for each unitary increase in the percentage of ABGs with hyperoxemia. Similarly, the time of exposure to hyperoxemia before the diagnosis of VAP, the prevalence of hyperoxia + hyperoxemia, as well as the daily excess O_2_, were independently associated with the risk of VAP (Table 6). Moreover, patients in the highest tertile of daily excess O_2_ showed a 4.3 times greater adjusted risk of developing VAP as compared to those in the lowest tertile. The total amount of excess O_2_ received before the diagnosis of VAP was not significantly associated with the onset of VAP, nor were the prevalence of hyperoxemia or hyperoxia + hyperoxemia in the first 3 days of mechanical ventilation and the duration of hyperoxemia in the first 3 days.

**TABLE 6:** Binomial logistic regression models for the association between hyperoxemia and VAP.

	Odds ratio (95% confidence interval)	*p*
**Model 1**		
Hyperoxemia, % of ABGs (before VAP)	1.033 [1.006–1.061]	0.015
Mean PaO_2_/FiO_2_ (mmHg)	0.983 [0.973–0.993]	0.001
Prolonged prone positioning (yes/no)	3.089 [1.228–7.767]	0.017
NMBA (days before VAP)	0.919 [0.814–1.037]	0.170
BMI (kg/m^2^)	1.031 [0.977–1.088]	0.266
RBCs before VAP (number of units)	1.014 [0.880–1.169]	0.843
**Model 2**		
Time of exposure to hyperoxemia, hours/day before VAP	1.108 [1.018–1.206]	0.018
Mean PaO_2_/FiO_2_ (mmHg)	0.982 [0.973–0.992]	<0.001
Prolonged prone positioning (yes/no)	3.040 [1.178–7.846]	0.022
NMBA (days before VAP)	0.908 [0.804–1.025]	0.118
BMI (kg/m^2^)	1.039 [0.985–1.097]	0.160
RBCs before VAP (number of units)	0.994 [0.861–1.147]	0.931
**Model 3**		
Hyperoxia + Hyperoxemia, % of ABGs (before VAP)	1.038 [1.003–1.075]	0.035
Mean PaO_2_/FiO_2_ (mmHg)	0.993 [0.985–1.002]	0.122
Prolonged prone positioning (yes/no)	3.138 [1.244–7.920]	0.015
NMBA (days before VAP)	0.925 [0.822–1.042]	0.199
BMI (kg/m^2^)	1.030 [0.976–1.087]	0.287
RBCs before VAP (number of units)	1.017 [0.885–1.169]	0.809
**Model 4**		
Daily excess O2 (L/day)	1.001 [1.000–1.001]	0.007
Mean PaO_2_/FiO_2_ (mmHg)	0.988 [0.980–0.997]	0.006
Prolonged prone positioning (yes/no)	3.102 [1.193–8.064]	0.020
NMBA (days before VAP)	0.900 [0.796–1.018]	0.095
BMI (kg/m^2^)	1.016 [0.962–1.073]	0.562
RBCs before VAP (number of units)	1.045 [0.906–1.206]	0.543
**Model 5**		
Daily excess O2 (L/day)		
First tertile (≤1,136)	Reference	*p* for trend 0.014
Second tertile (≤1,561)	1.712 [0.672–4.363]	0.260
Third tertile (>1,561)	4.332 [1.595–11.767]	0.004
Mean PaO_2_/FiO_2_ (mmHg)	0.988 [0.980–0.997]	0.006
Prolonged prone positioning (yes/no)	2.666 [1.035–6.865]	0.042
NMBA (days before VAP)	0.913 [0.811–1.028]	0.134
BMI (kg/m^2^)	1.024 [0.970–1.081]	0.389
RBCs before VAP (number of units)	1.044 [0.905–1.204]	0.554

All variables were calculated before the onset of VAP.

Model 1: This model was statistically significant with a χ^2^ (df 6) = 23.035 and *p* = 0.001. This model explains 21.1% (Nagelkerke *R*^2^) of variance for the outcome VAP and correctly classifies 66.4% of cases.

Model 2: This model was statistically significant with a χ^2^ (df 6) = 27.746 and *p* = 0.001. This model explains 24.9% (Nagelkerke *R*^2^) of variance for the outcome VAP and correctly classifies 65.7% of cases.

Model 3: This model was statistically significant with a χ^2^ (df 6) = 21.622 and *p* = 0.001. This model explains 19.9% (Nagelkerke *R*^2^) of variance for the outcome VAP and correctly classifies 67.2% of cases.

Model 4: This model was statistically significant with a χ^2^ (df 6) = 25.094 and *p* < 0.001. This model explains 22.8% (Nagelkerke *R*^2^) of variance for the outcome VAP and correctly classifies 69.4% of cases.

Model 5: This model was statistically significant with a χ^2^ (df 6) = 25.784 and *p* = 0.001. This model explains 23.3% (Nagelkerke *R*^2^) of variance for the outcome VAP and correctly classifies 67.9% of cases.

## Discussion

This retrospective single-centre study on 134 mechanically ventilated patients with SARS-CoV-2 pneumonia showed that: first, the dose of supplemental O_2_ administered was often excessive in comparison to the oxygenation target indicated by current guidelines and the exposure to hyperoxemia was frequent; second, in most cases of hyperoxemia the FiO_2_ was not varied, adjustments in O_2_ dose were made more frequently in presence of higher PaO_2_ or higher initial FiO_2_; third, the exposure to hyperoxemia was independently associated with higher risks of ICU-mortality and VAP.

Our data are consistent with those of previous studies showing that clinicians generally tolerate higher PaO_2_ values than those commonly recommended. In a retrospective study in critically ill mechanically ventilated patients, de Graaf et al. showed a prevalence of hyperoxemia (PaO_2_ > 120 mmHg) of 22% and adjustments in the FiO_2_ were rarely made, especially if this was ≤40% (21). Suzuki et al. calculated that an excess O_2_ dose of 3,472 L per patient was administered on average during mechanical ventilation and no change in the FiO_2_ was made in most cases of hyperoxemia if the initial level was 30–40% (22). In a recent study in COVID-19 mechanically ventilated patients, the prevalence of hyperoxemia in the ICU using a standard oxygenation protocol was 75.9% in the first day (23). Similarly in our study, the FiO_2_ was more likely to be reduced in presence of more severe hyperoxemia, whereas PaO_2_ values of 100–119 mmHg were accepted in more than 80% of cases. The initial FiO_2_ also determined the clinicians’ behavior against hyperoxemia: if this was <40% no change was made in almost 90% of cases, while an initial FiO_2_ ≥ 80% was corrected in more than 60% of cases.

A possible explanation of this too liberal attitude toward O_2_ therapy is a lack of perception of the risks associated with hyperoxemia. A survey by Helmerhorst et al. showed that most clinicians recognize the potential deleterious effects of a prolonged exposure to excessive O_2_ concentrations, including hyperoxia-induced lung injury, and show little tolerance toward even mild hyperoxemia (24). Nonetheless, a large proportion of their ICU patients was exposed to higher arterial O_2_ levels than self-reported target ranges (24). These data suggest that, in actual clinical practice, clinicians tend to tolerate a certain degree of hyperoxia, as perceived as a safety buffer against hypoxemia. In the context of the COVID-19 pandemic, such a liberal attitude may be partly justified by a greater fear against episodes of hypoxemia in a clinical scenario in which a ready accessibility to the patient is not always guaranteed due to the isolation precautions, and an SpO_2_ of 98–100% on the monitor could appear more reassuring than an SpO_2_ of 90–92%.

As a matter of fact, O_2_ toxicity and hyperoxia-induced lung injury are relatively slow processes in comparison to more acute conditions that can rapidly induce pulmonary oedema, respiratory failure and shock (such as aspiration pneumonia or sepsis-induced ARDS). A continuous exposure to hyperoxia for days/weeks can lead to diffuse alveolar damage, respiratory failure and high risk of death, while prolonged exposures to sub-lethal O_2_ doses generally induce pulmonary fibrosis (1). In healthy volunteers, breathing 95% O_2_ for 17 h caused a significant alveolar-capillary leak, due to a progressive destruction of the alveolar-capillary membrane, alveolar hemorrhage, formation of microthrombi, and intrapulmonary shunt (25). Moreover, oxidative stress compromises the surfactant system, thus causing atelectasis and reduction in lung compliance (26). Patients with ARDS may be particularly susceptible to O_2_ toxicity, since the ongoing inflammatory process may already compromise their adaptive and anti-oxidant capacities. Moreover, hyperoxia may aggravate and predispose the lung to the deleterious effects of positive pressure ventilation (5).

A recent meta-analysis of RCTs showed that the use of higher oxygenation targets in the critically ill may increase mortality, even if with a very-low level of evidence (27). The LOCO_2_ trial failed to prove the safety and efficacy of a more restrictive oxygen therapy in patients with ARDS (11), however, these results cannot be conclusive. Similarly, the multi-center HOT-ICU trial did not show any difference in 90-day mortality with a lower oxygenation target as compared to higher PaO2 target in patients with acute hypoxemic respiratory failure (28). Our study showed that the exposure to hyperoxemia in mechanically ventilated patients with SARS-CoV-2 pneumonia may be associated with higher ICU mortality, independently of other risk factors such as the mean PaO_2_/FiO_2_, SOFA score at admission and age. Moreover, a higher prevalence of hyperoxemia, as well as an excess O_2_ administration, was independently associated with a greater risk of VAP.

Ventilator-associated pneumonia is the most common ICU-acquired infection among mechanically ventilated patients, leading to higher mortality, longer need for mechanical ventilation, and an increase in healthcare cost (18). For the implementation of effective preventive strategies, it is crucial to gain a deep understanding of the pathophysiology and risk factors for VAP. There is ample evidence that a long-term exposure to hyeroxia can impair pulmonary innate immunity and bacterial phagocytosis capacity (29). In a retrospective study on mechanically ventilated critically ill patients, the presence of hyperoxemia (defined as PaO_2_ > 120 mmHg) at ICU-admission and the number of days of exposure to hyperoxemia were independently associated with the onset of VAP (6). Preclinical studies in animal models also supported a role of hyperoxia in the pathogenesis of VAP. Entezari et al. showed that a prolonged exposure to hyperoxia can compromise the ability of alveolar macrophages to phagocytose Pseudomonas Aeruginosa (30) and increased mortality in infected mice (31). Hyperoxia increased mortality in mice with Acinetobacter pneumonia, in which the administration of procysteine was able to improve survival by increasing the phagocytic activity of alveolar macrophages under hyperoxic conditions (32). By applying a too liberal O_2_ therapy, we may be losing the opportunity to control for a significant risk factor for VAP, a complications that occurred in almost 50% of patients in our cohort.

In our study, the use of prolonged prone positioning (sessions of up to 36 consecutive hours) was also associated with an increased risk of VAP. From a theoretical point of view, prone positioning could prevent the onset of VAP by facilitating the drain of respiratory secretions and limiting ventilator-induced lung injury, although most clinical studies showed no significant impact on the actual incidence of VAP (33). Several factors may explain the association observed, including the need for prolonged administration of NMBA and deep sedation, the increase in abdominal pressure with a higher risk of aspiration of gastric content, the need to limit the dose of enteral nutrition. ICU-acquired weakness is a frequent complication in COVID-19 patients, especially in those who received higher doses of NMBA and sedatives and lower caloric/protein intake, and may be responsible for a longer duration of mechanical ventilation and prolonged exposure to the risk of VAP (34).

Our study has several limitations. First, the retrospective design that does not allow to define a cause-effect relationship between the exposure to hyperoxia and mortality or VAP, but only enables to describe associations. Second, the relatively low sample size and the involvement of a single center, which limits the generalizability of our results. The sample size limited the number of confounders that could be included in multivariate regression models, since the inclusion of too many independent variables would lead to a mathematically unstable outcome (20). Third, we based our analysis on ABG data that can only provide a partial picture of the exposure to hyperoxemia, limited to the moment in which the ABG was made. Unfortunately, we could not collect SpO_2_ data, which could have been useful for a more continuative evaluation of the oxygenation status and the responses of clinicians to SpO_2_ values above the target. Fourth, the calculation of the “ideal FiO2” and “excess O2” can only provide an imprecise estimate of the amount of O2 administered in excess. In fact, factors such as PEEP, prone positioning or use of iNO will determine the most appropriate FiO2 of the patient by influencing gas exchange: therefore, calculating the ideal FiO2 merely on the basis of the PaO2/FiO2 ratio may be reductive. Despite its limitations, to the best of our knowledge, this is the first study that describes the prevalence of hyperoxemia in mechanically ventilated COVID-19 patients and explores the potential effects of excess O_2_ doses on outcome. Future larger studies are needed to confirm our findings.

## Conclusion

In mechanically ventilated patients with SARS-CoV-2 pneumonia admitted to our ICU, the administration of O_2_ was often excessive in comparison to the PaO_2_ target indicated by the guidelines, and the exposure to hyperoxemia was frequent. In addition, most episodes of hyperoxemia were not followed by a reduction in the FiO_2_; changes were made more frequently in the presence of higher PaO_2_ or higher initial FiO_2_. The prevalence of hyperoxemia was independently associated with a greater risk of ICU-mortality, as well as with a greater risk of developing VAP. The retrospective nature of our study does not allow to draw conclusions on a possible cause-effect relationship between the exposure to excessive amounts of O_2_ and outcome. However, these data add to the ample literature that warns against the possible deleterious effects of a too liberal O_2_ therapy. In the absence of strong evidence of the safety of hyperoxemia in critically ill patients (in particular in those with ARDS and SARS-CoV-2 pneumonia), more efforts should be made to avoid the exposure to excessive amounts of supplemental O_2_. Further studies are needed to define the best oxygenation target for this patient category.

## Data availability statement

The raw data supporting the conclusions of this article will be made available by the authors, without undue reservation.

## Ethics statement

The studies involving human participants were reviewed and approved by Comitato Etico Regionale Marche. Written informed consent for participation was not required for this study in accordance with the national legislation and the institutional requirements.

## Author contributions

ED contributed to the study design, analysis, and interpretation of data and drafted the manuscript. EC, AC, EA, and AD contributed to the study design and interpretation of the data and revised the manuscript critically. GM, SV, RG, RD, and CS participated in the acquisition and analysis of the data. All authors have agreed both to be personally accountable for the author’s own contributions and to ensure that questions related to the accuracy or integrity of any part of the work, even ones in which the author was not personally involved, are appropriately investigated, resolved, and the resolution documented in the literature and approved the submitted version of the manuscript.

## Conflict of interest

The authors declare that the research was conducted in the absence of any commercial or financial relationships that could be construed as a potential conflict of interest.

## Publisher’s note

All claims expressed in this article are solely those of the authors and do not necessarily represent those of their affiliated organizations, or those of the publisher, the editors and the reviewers. Any product that may be evaluated in this article, or claim that may be made by its manufacturer, is not guaranteed or endorsed by the publisher.
